# Phenotype of higher post-load insulin response as a predictor of all-cause mortality and cardiovascular mortality in the Chinese non-diabetic population

**DOI:** 10.1186/s13098-022-00786-0

**Published:** 2022-01-28

**Authors:** Xiaoxia Shen, Siyao He, Jinping Wang, Xin Qian, Hui Wang, Bo Zhang, Yanyan Chen, Hui Li, Guangwei Li

**Affiliations:** 1 Center of Endocrinology, Fuwai Hospital, Chinese Academy of Medical Sciences, No. 167 North Lishi Road, Xicheng District, Beijing, 100037 China; 2 Department of Cardiology, Da Qing First Hospital, No. 9 Zhongkang Street, Saltu District, Daqing, 163411 Heilongjiang China; 3 Department of Endocrinology, China-Japan Friendship Hospital, No 2, East Yinghua Road, Chaoyang District, Beijing, 100029 China

**Keywords:** Insulin response, All-cause death, CVD death

## Abstract

**Aim:**

This study aimed to assess whether a higher insulin response increased the long-term risk of mortality in a non-diabetic population.

**Methods:**

A total of 446 people with normal glucose tolerance (NGT) or impaired glucose tolerance (IGT) who participated in the Da Qing Diabetes Study, were stratified into quartiles subgroups according to their baseline insulin area under the curve (AUC) during oral glucose tolerance test, defined as Q1, Q2, Q3 and Q4. The participants were followed from 1986 to 2016 to assess the risk of death in association with the magnitude of post-load insulin response.

**Results:**

Over 30 years, the rates of all cause death were 9.94, 14.81, 15.02, and 17.58 per 1000 person-years across the four groups respectively. The rate for cardiovascular disease (CVD) death was 5.14, 6.50, 6.80 and 10.47 per 1000 person-years. Compared with Q1, the risk of all-cause death was significantly higher in participants in Q4 (HR = 2.14, 95% CI 1.34–3.42), Q3 (HR = 1.94, 95% CI 1.20–3.14), and Q2 group (HR = 1.70, 95% CI 1.06–2.74). In the Fine-Gray model with non-CVD death as competing risk, the increased insulin AUC were also significantly associated with the CVD death (Q4 vs Q1, HR = 2.04, 95% CI 1.10–3.79). In the fractional polynomial regression analysis, a nonlinear association between insulin AUC and all-cause and CVD death was demonstrated. In addition, insulin AUC was associated with a progressively higher risk of all-cause death and CVD death (fractional power 3, P < 0.001).

**Conclusion:**

A higher post-load insulin response was significantly associated with a long-term increased risk of all-cause and CVD deaths in the Chinese non-diabetic population. It suggests that people featured by this phenotype is a potential important target for further intervention.

## Introduction

Several studies had suggested that a higher blood insulin concentration in non-diabetic people may have contributed to the increased risk of death. However, the conclusion remained uncertain. The 22-year follow-up investigation of Helsinki Policemen Study showed that the highest quintile of the area under the insulin response curve among middle-aged men with normal blood glucose levels, compared with the combined 4 lower quintiles, was associated with a 2-fold higher risk of stroke, but this association was not independent of other risk factors such as upper body obesity, hypertension, and smoking [1]. In line with that result, an 11-year follow-up investigation of the Paris Prospective Study demonstrated that fasting plasma insulin level was an independent predictor of cardiovascular disease death after adjusting for overt diabetes [2]. Interestingly, the results varied in the 15-year follow-up investigation of the same study, wherein the 2-h post-load plasma insulin level was a significant predictor of CVD-related death, while levels of blood glucose were not a significant predictor after the adjustment of plasma insulin levels. The author addressed that insulin response to oral glucose tolerance test (OGTT) requires further investigation as a potential risk factor for coronary artery disease and a potential target for further intervention [3].

A 30-year follow-up of The Da Qing Diabetes Prevention Study showed that prevention or delay of diabetes can extend to the decrease of diabetes-related CVD events by 26% and a 1.44-year longer life expectancy [4]. However, it is not clear whether the baseline high post-load insulin response is correlated with the risk of mortality in the non-diabetic population. The present analysis aimed to investigate whether the phenotype of a higher post-load insulin response in non-diabetic population (including people with NGT and IGT) predicted an increased long-term risk of all-cause and CVD mortality.

## Participants and methods

In total, 446 non-diabetic subjects, including 256 with impaired glucose tolerance (IGT) and 190 with normal glucose tolerance (NGT) identified by standard OGTT in 1986, were included in the present study. All the recruited participants had been part of the original Da Qing Diabetes Prevention Study. Details of the study design, methods, and population have been reported previously [5–7]. Briefly, in 1986, a total of 110,660 people (accounting for 50% of the adult residents) in Daqing, China, participated in a screening program for diabetes. Among them, 576 subjects with IGT and age- and sex-matched 519 subjects with NGT were identified based on the 1985 World Health Organization (WHO) criteria. In the present analysis, 19 IGT subjects with fasting plasma glucose greater than 7.0 mml/L were excluded in order to follow the current diabetes criteria (WHO, 1999). Because the focus of this study was the association of initial insulin response with the risk of death, only people with full data of plasma glucose, insulin levels at fasting and post-glucose load 1 hour and 2 hours were recruited. The participants were stratified into four subgroups (Quartiles) according to their baseline insulin AUC during a 75-g glucose load OGTT in 1986, defined as Q1, Q2, Q3 and Q4 groups. The insulin AUC during OGTT was calculated through trapezoidal estimation of the plasma insulin level at 0 min, 60 min, and 120 min with the following formula: insulin AUC=fasting insulin (mU/L)/2 + insulin 1h (mU/L) + insulin 2h (mU/L)/2. The date and cause of death were verified by review of patients’ medical records, death certificates, or both. Details of follow-up and data collection were reported elsewhere [8].

### Statistical analysis

Data are presented as mean (±SD) or count (percentage). Descriptive statistics were used to compare groups of participants with different levels of insulin AUC. All-cause death and CVD death rates in the four quartiles of the insulin AUC groups were estimated as the number of deaths divided by the number of person-years. Confidence intervals (CIs) were calculated using Fisher’s exact method. The incidences of all-cause and CVD mortality were compared among groups using Kaplan-Meier curves. The proportional hazards assumption was assessed using Schoenfeld's residuals test. If the slope of the fitted curve is not different from zero, or the P value of ZPH test is greater than 0.05, it is considered to meet the proportional hazard assumption. The hazard ratios (HRs) were obtained using the Cox proportional hazards model after adjusting for related confounders. The Fine and Gray analysis was used to evaluate if the effect of insulin AUC was due to CVD death.

A fractional polynomial regression model was used to explore the nonlinear association between insulin AUC and death. Before multiple analysis, variance inflation factor (VIF) test was used to determine multicollinearity [9]. Only variables determined without multicollinearity (VIF values<10) were included in the model. Two-tailed P values < 0.05 were considered statistically significant. The multiple imputation method was used to impute the missing data. Statistical analyses were performed using SAS for Windows, version 9.4 (SAS Institute Inc, Cary NC, USA) and Stata SE (version 16, Stata Corp).

## Results

Across the four groups with increased levels of insulin AUC at baseline, the baseline fasting and postprandial plasma glucose, triglyceride, cholesterol levels and blood pressure gradually increased. The insulin AUC increased drastically from 75.23, 129.61, 179.06 to 309.85 mU/L, with the BMI increasing from 22.83, 23.99, 25.63 to 27.29 kg/m^2^. The number of males and smokers in Groups 3 and 4 was significantly lower than that in Groups 1 and 2 (Table 1).

**Table 1:** Baseline characteristics of the study participants with different levels of insulin area under the curve (insulin-AUC) during oral glucose tolerance test

	Q1 (n = 111)	Q2 (n = 109)	Q3 (n = 112)	Q4 (n = 114)	P value
Age (years)	43.45 ± 7.97	42.71 ± 8.94	43.32 ± 8.96	45.77 ± 8.18	0.0390
Sex (% men)	54.05	59.63	47.32	41.23	0.0356
Current Smoker (%)	45.95	47.71	30.36	36.84	0.0276
BMI (kg/m^2^)	22.83 ± 3.00	23.99 ± 3.07	25.63 ± 3.30	27.29 ± 3.81	< 0.0001
FPG (mmol/L)	4.92 ± 0.71	5.15 ± 0.84	5.21 ± 0.72	5.34 ± 0.79	0.0007
2hPG(mmol/L)	6.19 ± 2.04	7.13 ± 2.37	7.50 ± 2.03	8.22 ± 1.58	< 0.0001
SBP (mmHg)	121.9 ± 20.3	127.2 ± 24.3	130.8 ± 20.0	134.4 ± 22.0	0.0002
DBP (mmHg)	81.38 ± 12.3	85.24 ± 17.1	87.10 ± 12.3	87.79 ± 13.1	0.0025
CHO (mmol/L)	4.88 ± 1.34	4.82 ± 0.94	5.10 ± 1.16	5.25 ± 1.14	0.0481
TG (mmol/L)	1.33 ± 1.35	1.19 ± 0.62	1.87 ± 1.82	2.12 ± 2.01	0.0004
FINS (mU/L)	14.65 ± 10.2	15.46 ± 7.1	24.01 ± 16.2	33.02 ± 19.0	< 0.0001
1hINS (mU/L)	54.98 ± 38.0	85.88 ± 17.9	116.28 ± 23.0	199.38 ± 69.5	< 0.0001
2hINS (mU/L)	43.86 ± 22.3	71.99 ± 32.4	101.56 ± 47.0	187.93 ± 79.0	< 0.0001
Insulin-area (mU/L)	75.23 ± 19.4	129.61 ± 14.3	179.06 ± 16.4	309.85 ± 82.6	< 0.0001

BMI, body mass index; FPG, fasting plasma glucose; 2hPG, 2-h plasma glucose; SBP, systolic blood pressure; DBP, diastolic blood pressure; CHO, Cholesterol, TG, triglyceride, FINS, fasting insulin level; 1hINS, 1-h insulin level; 2hINS, 2-h insulin level; Insulin-area: Insulin area under the curve; Q1, Q2, Q3 and Q4 were quartile groups of baseline insulin area under the curve

During the 30-year follow-up period, all-cause death rates of the four groups were 9.94 (95% CI 6.32–13.56), 14.81 (95% CI 10.27–19.34), 15.02 (95% CI 10.48–19.57), and 17.58 (95% CI 12.55–20.60) per 1000 person-years, respectively. The corresponding rates for CVD death were 5.14 (95% CI 2.54–7.74), 6.50 (95% CI 3.50–9.50), 6.80 (95% CI 3.74–9.85), and 10.47 (95% CI 6.59–14.35) (Table 2).

**Table 2:** All-cause and CVD death rates in groups with different levels of baseline insulin-AUC over 30-year follow-up

	Q1(n = 111)	Q2(n = 109)	Q3(n = 112)	Q4(n = 114)
*All-cause death*				
Number	29	41	42	47
Follow-up person-years	2917	2769	2795	2674
Incidence per 1000 person years (95% CI)	9.94 (6.32–13.56)	14.81 (10.27–19.34)	15.02 (10.48–19.57)	17.58 (12.55–22.60)
*CVD deaths*				
Number	15	18	19	28
Follow-up person-years	2917	2769	2795	2674
Incidence per 1000 person years (95% CI)	5.14 (2.54–7.74)	6.50 (3.50–9.50)	6.80 (3.74–9.85)	10.47 (6.59–14.35)

Q1, Q2, Q3, and Q4 were quartile groups of baseline insulin-AUC; CI, confidence interval; HR, hazard ratio

**Fig. 1:**
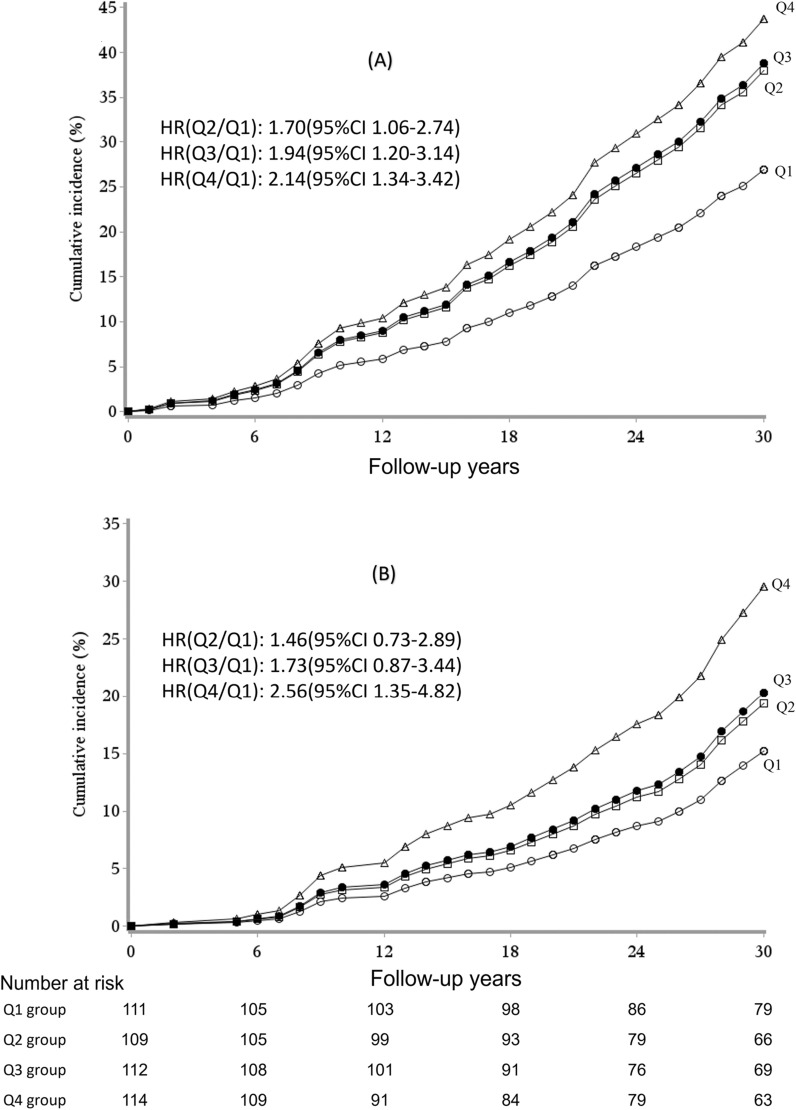
Cumulative incidence and hazard ratios (HRs) of all-cause (**A**) and CVD death (**B**) in 2016 among non-diabetes participants at baseline with different levels of insulin area under curve. HRs was adjusted for age,sex and smoking. The triangles represent Q4, black circles represent Q3, squares represent Q2 and open circles represent Q1

After adjusted for age, sex and smoking status, Cox proportional hazards analysis showed that compared with Q1, the risk of all-cause death was significantly higher in participants in Q4 (HR=2.14, 95% CI 1.34–3.42), Q3 (HR=1.94, 95% CI 1.20–3.14), and Q2 (HR=1.70,95% CI 1.06–2.74). As for CVD death risk, participants in Q4 showed a higher risk than those in Q1 (HR=2.56, 95% CI 1.35–4.82) (Fig. 1). Multivariable analysis models showed after adjusting for age, sex, smoking, plasma total cholesterol, baseline BMI, systolic blood pressure (SBP) and 2-h postprandial blood glucose, the risks of all-cause death in those in Q4 and Q3 were significantly higher than those in Q1 (HR 1.90,95% CI 1.12–3.23 and HR 1.80,95% CI 1.09–2.97). In addition, 2-h postprandial blood glucose independently associated with the risk of all-cause death (HR 1.08, 95% CI 1.00–1.17) (Table 3).

**Table 3:** Impact of insulin AUC and 2hPG on risk of occurrence of all-cause death over 30-year follow-up

Variables	HR	95%CI		P value
Age (years)	1.09	1.07	1.11	< 0.0001
Sex (M)	1.56	1.08	2.26	0.0172
Current Smoker (yes = 1)	1.76	1.26	2.46	0.001
BMI (kg/m^2^)	0.96	0.91	1.01	0.1039
Total Cholesterol (mmol/L)	1.08	0.94	1.25	0.2967
SBP (mmHg)	1.01	1.01	1.02	< 0.0001
2hPG (mmol/L)	1.08	1.00	1.17	0.0481
Q2/Q1	1.55	0.95	2.52	0.0767
Q3/Q1	1.80	1.09	2.97	0.0212
Q4/Q1	1.90	1.12	3.23	0.0166

*BMI* body mass index, *SBP* systolic blood pressure, *2hPG* 2-h plasma glucose; Q1, Q2, Q3 and Q4 were quartile groups of baseline insulin-AUC; CI, confidence interval; HR, hazard ratio

**Figure. 2:**
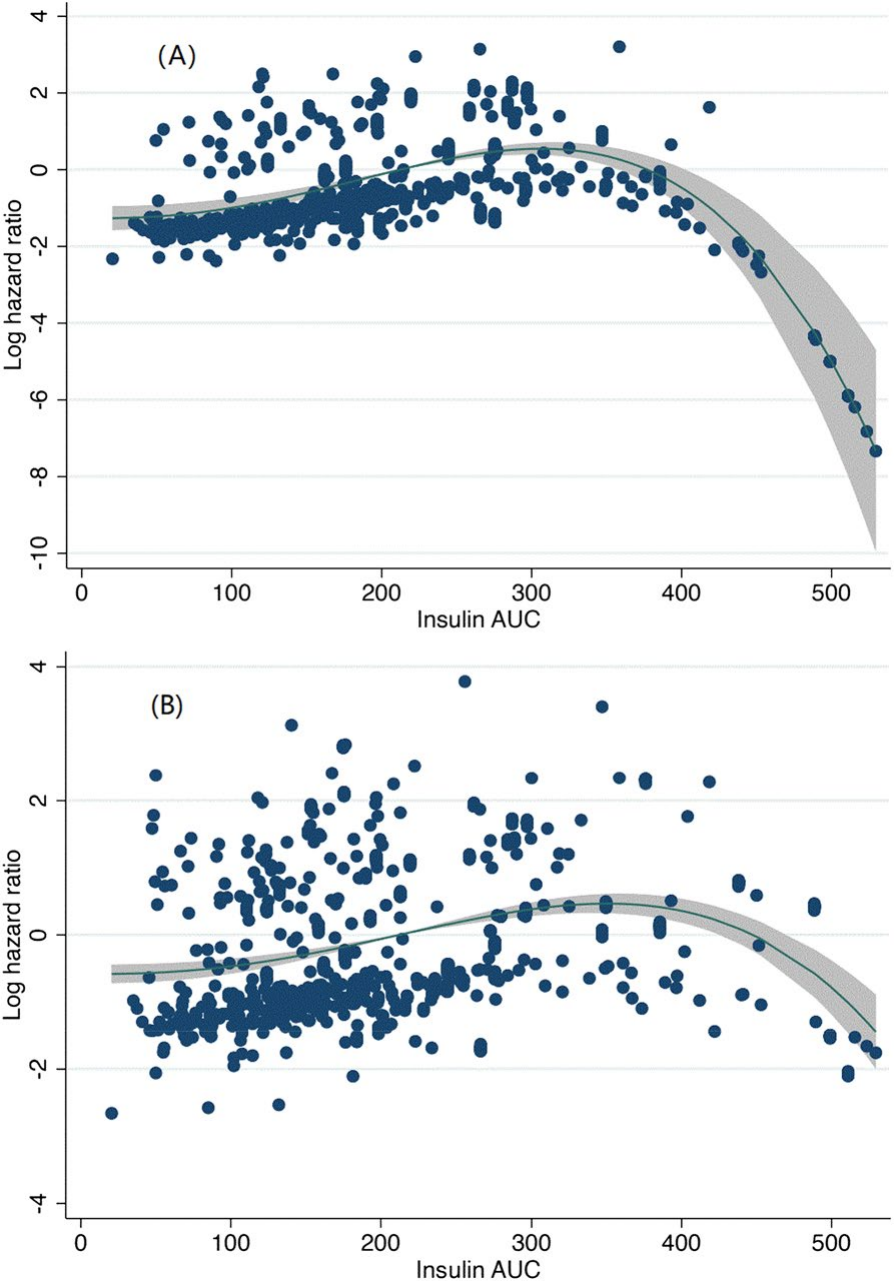
The figure represents the nonlinear association of insulin AUC with all-cause death (**A**) and CVD death (**B**). Log hazard ratios represent risk of all-cause death with the increase of insulin AUC. Adjusted for: Age, sex, smoking, BMI, SBP, plasma total cholesterol and 2-h postprandial blood glucose. The black line represents the hazard ratio and the shaded area, the 95% confidence interval

In the Fine-Gray model analysis with non-CVD death as competing risk, the increased insulin AUC were also significantly associated with the CVD death (Q4 vs Q1, HR=2.04, 95% CI 1.10–3.79) after adjusted for age, sex and smoking status, whereas in the model with CVD death as competing risk, the result was HR=1.39 (95% CI 0.69–2.79).In the fractional polynomial regression analysis, after fully adjusting for age, sex, smoking, plasma total cholesterol, baseline BMI,SBP and 2-h postprandial blood glucose, a nonlinear association between insulin AUC values and death risk was demonstrated. Insulin AUC were associated with a progressively higher risk of all-cause death and CVD death (fractional power 3, P < 0.001). The results in Fig. 2 showed that it was the moderate increase of insulin AUC (from the mean insulin AUC of 75 mU/L in Q1 to the mean insulin AUC of 179 mU/L in Q3) caused the increased risk of death drastically.

## Discussion

Many studies support the notion that CVD is related to insulin resistance [10–12]. However, results from different studies are controversial. In the 8.6 years’ follow-up of the Prospective Study of Pravastatin in the Elderly (PROSPER), higher Homeostasis Model Assessment of Insulin Resistance (HOMA-IR) was not associated with all-cause and CVD mortality or fatal/non-fatal CVD [13]. There is a paucity of evidence whether a higher post-load insulin response induced by insulin resistance is a risk factor for CVD mortality in people with normal glucose status and IGT.

In the present study, we found that non-diabetic Chinese people with higher insulin AUC in response to OGTT, an indicator of higher insulin demands for maintaining glucose metabolism, had significantly higher rates of all-cause and CVD mortality during the subsequent 30 years. Multiple regression analysis further confirmed that baseline insulin AUC significantly predicted these risks. A more interesting finding was that the predictive effect of insulin AUC on mortality coexisted with a significant association between death and 2-hour post load glucose levels of the participants after controlling for the influence of traditional risk factors, including obesity, age, sex, smoking, blood pressure and cholesterol. After adjusting for 2-h postprandial blood glucose, the HR showed an increasing trend across the quartile groups of insulin AUC, the P value changed from 0.08, 0.02 to 0.017.

It indicated that a higher post-load insulin response in non-diabetic participants contributed to increased risk of mortality independent of other cardiovascular risk factors. This finding highlighted that a strategy only focusing on blood glucose control but ignoring role of the higher post-load insulin response may not reduce the overall risks of death.

It was also found that the mortality rates in people with IGT were significantly higher than those with NGT. A possible reason is that the insulin levels in IGT people are much higher than people with NGT at the presence of a mild hyperglycemia. Therefore, to identify those non-diabetic subjects with higher post-load insulin response should be included in the strategies of lowing all-cause and CVD mortality. Accordingly, to eliminate these clustered risk factors, a package plan to deal with them in a coordinated and balanced manner is warranted.

Why hyperinsulinemia increased the risk of death in the non-diabetic population? Higher post-load insulin response was a manifestation of hyperinsulinemia. Clinically, an accepted reason is that the hyperinsulinemia in the non-diabetic subjects triggered the onset of diabetes, and then the development to diabetes exacerbated the high risk of any death, especially CVD death. Findings in basic research also provided some insights into understanding the complexity between hyperinsulinemia and CVD risk. Chronic hyperinsulinemia causes coronary vasoconstriction by increasing the release of endothelin-1(ET-1) and sympathetic nerve activity and by reducing endothelial response to vasodilators [14]. It is also associated with several dysregulations of coagulation and fibrinolysis [15]. Nolan et al proposed a more convincing concept of insulin mediated metabolic stress for conditions related to insulin resistance [16]. It stated that the insulin resistance had conferred protection against the nutrient overload and metabolic stress by limiting glucose flux into the cell. However, the iatrogenic hyperinsulinemia to override insulin resistance may also override the defence mechanism because the tissues will no longer gain protection from excess nutrient entry. Recently, this idea was supported by H. Kolb’s team, wherein they stated that too high systemic insulin levels are detrimental for body functions [17]. For non-diabetic, the higher post-load insulin response for maintaining normal blood glucose level may cause insulin-induced metabolic stress in target organs, thereby increasing the risk of death. This means that higher post-load insulin response is harmful to the body even in the non-diabetic population and should therefore be avoided. Most people with IGT and higher insulin response in the present study have deterioration of diabetes during the 30-year follow-up period. Therefore, findings in this non-diabetic population, especially both higher post load 2h glucose and higher insulin response were significantly associated with the increased risk of all-cause death and CVD death, may also have some implications for people with diabetes.

The advanced, long-term type 2 diabetes with poor glycaemic control is often characterised by high blood glucose and lowering insulin levels and severe insulin resistance. In epidemiological studies of type 2 diabetes, it has been consistently observed that the addition of insulin to the treatment regimen or the intensification of insulin treatment may significantly improve the glucose control, but at the same time it had to pay the price of hyperinsulinemia, which may lead to insulin-induced metabolic stress in the heart. It was reported that over-nutrition-induced metabolic stress is harmful to the heart [18–20]. Like iatrogenic hyperinsulinemia, the higher post-load insulin response in patients with type 2 diabetes may lead to insulin-induced metabolic stress in the heart (metabolic cardiomyopathy), followed by increased risk of cardiac failure and arrhythmias [21–23]. Unfortunately, hyperinsulinemia is almost inevitable for better glucose control because more than 80% of type 2 diabetes patients are resistant to insulin. Even worse, it is possible that this insulin resistance and hyperinsulinemia status may exist for lifetime. Thus, in the real-word clinical practice, how to balance the benefits of better blood glucose control and adverse effects of exogenic hyperinsulinemia in patients with type 2 diabetes has become a big challenge.

For alleviating higher insulin response, both lifestyle modification and using medications, such as Glucagon-like peptide-1 (GLP-1) receptor agonists or metformin, should not be ignored. The present data showed that the baseline insulin AUC sequentially increased with the increase in BMI across the four groups, suggesting that insulin-induced harm is more likely to occur in overweight/obese people. Therefore, losing weight and improving insulin sensitivity may be good ways to avoid higher insulin response and prevent the development of diabetes.

Our study has several strengths. First, a standard OGTT was conducted at baseline; thus, plasma insulin AUC over 2 hours could be calculated. Second, very few people migrated away from the city, with a minimum rate of loss to follow-up. Third, the participants were followed up for 30 years, which was an optimal duration for patients to develop life-threatening complications such as CVD and stroke.

The limitations of the study should also be acknowledged. The sample size was relatively small. Furthermore, we had no systemic data of medications for the treatment of hypertension and hyperlipidaemia over the 30-year follow-up period; therefore, we are unable to evaluate the impact of these factors on the risk of mortality in the study population. Finally, the insulin level determined by radioimmunoassay (RIA) cannot completely exclude proinsulin, so the results of those people with extremely high insulin values may be affected. New researches using more advanced methods to measure insulin or real-world data may provide more information on this aspect.

## Conclusions

A higher insulin response to OGTT significantly associated with the long-term risk of all-cause and CVD mortality in a population of Chinese adults who were not diabetic at baseline. Our findings suggest that people featured by the phenotype of high insulin response to OGTT may be a potential important target for further intervention to reduce all-cause and CVD mortality.

## Data Availability

All data used to support the findings of this research are available on request from the corresponding author.
